# Temporal dynamics of daylight perception: Detection thresholds

**DOI:** 10.1167/jov.20.13.18

**Published:** 2020-12-29

**Authors:** Ruben Pastilha, Gaurav Gupta, Naomi Gross, Anya Hurlbert

**Affiliations:** 1 Neuroscience, Institute of Biosciences, Newcastle University, Newcastle upon Tyne, UK

**Keywords:** temporal illumination, detection thresholds, daylight perception, color constancy, chromatic adaptation, color vision, visual perception

## Abstract

Temporal changes in illumination are ubiquitous; natural light, for example, varies in color temperature and irradiance throughout the day. Yet little is known about human sensitivity to temporal changes in illumination spectra. Here, we aimed to determine the minimum detectable velocity of chromaticity change of daylight metamers in an immersive environment. The main stimulus was a continuous, monotonic change in global illumination chromaticity along the daylight locus in warmer (toward lower correlated color temperatures [CCTs]) or cooler directions, away from an adapting base light (CCT: 13,000 K, 6500 K, 4160 K, or 2000 K). All lights were generated by spectrally tunable overhead lamps as smoothest-possible metamers of the desired chromaticities. Mean detection thresholds (for 22 participants) for a fixed duration of 10 seconds ranged from 15 to 2 CIELUV ΔE units, depending significantly on base light CCT and with a significant interaction between CCT and direction of change. Cool changes become less noticeable for progressively warmer base lights and vice versa. For the two extreme base lights, sensitivity to changes toward neutral is significantly lower than for the opposite direction. The results suggest a “neutral bias” in illumination change discriminability, and that typical temporal changes in daylight chromaticity are likely to be below threshold detectability, at least where there are no concomitant overall illuminance changes. These factors may contribute to perceptual stability of natural scenes and color constancy.

## Introduction

Illumination makes objects visible by giving them light to reflect. Yet to what extent is the illumination itself visible to the human visual system? Here, we examine visual perception of the illumination through assessing sensitivity to temporal changes in illumination chromaticity.

The illumination in its complete form may be defined as a complex light field, varying in spectral irradiance over three-dimensional space, and resulting from a mixture of emissive light sources and indirect mutual surface reflections (Bloj, Kersten, & Hurlbert, 1999; Forsyth & Zisserman, 1989; Koenderink et al., 2007). Although the light emitted from traditional manmade sources tends to be largely static (Thorington, 1985), natural light is characteristically dynamic, changing in both spectral shape and overall irradiance over both short and long-time scales. This dynamic behavior results from temporal variations in the geometrical and spectral properties of both the direct light sources and indirect mutual reflections. For example, the illumination in forests may vary widely over space and time when the wind blows through a tree canopy, altering the way leaves both filter and reflect direct daylight. Other changes may occur due to cloud movement (Rahim, Baharuddin, & Mulyadi, 2004), changes in atmospheric turbidity and aerosols (Gueymard, 2005), and projection of shadows due to large terrain structures in the path of the solar beam. Yet the primary changes in natural light are the massive, gradual spectral variations caused by changes in solar elevation. These manifest as changes in both overall irradiance and correlated color temperature (CCT) occurring from dawn to dusk in the downwelling light that forms the global illumination at ground level (Judd et al., 1964; Lee, 1994; Spitschan et al., 2016).

Despite such large temporal changes in global illumination and the consequent changes in the reflected light from objects, the color appearance of objects tends to remain stable, through the perceptual phenomenon of color constancy (Hurlbert, 1998; Hurlbert, 2007; Radonjić, Cottaris, & Brainard, 2015). Color constancy has typically been measured by comparing the color appearance of surfaces under small sets of distinct static illumination conditions. Quantitative estimates of constancy vary substantially between studies, depending on physical properties of both the illuminations and surfaces under comparison (Hurlbert, 2019)**.** Given other evidence that human color vision is tuned to the spectral statistics of the natural environment (Foster, Amano, & Nascimento, 2006; Montagner et al., 2016; Webster & Mollon, 1997), it has been proposed that color constancy is optimized for natural illumination changes (Delahunt & Brainard, 2004; Pearce et al., 2014). The evidence for such optimization is mixed (Hurlbert, 2019). For example, asymmetric matching experiments – requiring observers to match the color appearance of a surface simulated under a test illumination with its appearance under a neutral illumination – have revealed better constancy for test illuminations along the blue-yellow axis (close to the daylight chromaticity locus) versus red-green axis (Daugirdiene et al., 2016; Worthey, 1985), whereas other experiments using similar paradigms suggest the opposite (e.g. Wan & Shinomori, 2018) or no difference between illuminations (Delahunt & Brainard, 2004). There is further evidence specifically for a “blue bias,” in two forms. Achromatic settings suggest that bluish hues are attributed to the illumination rather than surface reflectance (Weiss, Witzel, & Gegenfurtner, 2017), whereas illumination discrimination tasks (IDTs) indicate better constancy specifically for bluish changes in illumination along the daylight locus (Aston et al., 2019; Pearce et al., 2014) also supported by other evidence (Radonjić & Brainard, 2016). Other studies suggest that the blue bias might be part of a broader neutral bias, a tendency for the human visual system to assume a neutral daylight illumination (Aston et al., 2019). Thus, other regions of the daylight locus and color space must be tested more broadly and systematically to ascertain fully how color constancy depends on the reference chromaticity and direction of illumination change.

In addition, the visibility of changes in illumination itself is also understudied. Color constancy studies have rightly focused on surface color appearance, rather than illumination perception. Yet surface reflectance and illumination are inextricably linked in the light signal reflected to the eye. Therefore, it is important to disentangle perceptual responses to the two components. In many color constancy experiments, comparisons are made between two simultaneously presented, spatially separated illuminations, or between a single scene and a representation held in memory (as for achromatic settings; Foster, 2011). Where a change in illumination occurs over time between successively presented scenes, the sequence is typically discontinuous and artificial.

Typically, illumination changes are represented as step changes between individual static illuminations either with an abrupt transition (Barbur & Spang, 2008; Foster, Amano, & Nascimento, 2001; Lee, Dawson, & Smithson, 2012) or a separation interval between them (Aston et al., 2019; Pearce et al., 2014; Radonjić et al., 2016). Thus, in terms of perceptual sensitivity to temporal changes in illumination, it is possible to infer conclusions only about perceptually discontinuous changes, not about the smooth changes typically associated with natural illuminations.

To assess sensitivity to natural illumination changes, more naturalistic stimuli are required, but the few studies (Kong et al., 2019; Linnell & Foster, 1996; Nascimento et al., 1996; Walmsley et al., 2015) that use continuous changes are not specifically concerned with measuring detection thresholds.

For example, in the operational color constancy paradigm, observers are asked to discriminate between changes in scene chromaticity due to illumination versus surface reflectance changes (Craven & Foster, 1992; Foster, Craven, & Sale, 1992). The surface reflectance changes are simulated by spatially nonuniform chromaticity changes, with different surfaces in the scene changing in opposing chromatic directions. Illumination changes are simulated by a spatially uniform change, all surfaces changing in the same chromatic direction. For abrupt, discontinuous changes in time, observers can differentiate the two types of change. The relative invariance of spatial cone-excitation ratios under natural illumination changes is likely to underpin this differentiability (Foster & Nascimento, 1994). Indeed, further studies demonstrate that the larger the violation of spatial cone-excitation ratio invariance, the less likely observers are to attribute the change to a natural illumination change, even where the latter underlies the difference between two images (Nascimento & Foster, 1997). To determine whether discriminability between these types of change depends on the temporal profile of the change, Linnell and Foster (1996) applied continuous changes over finite time intervals and asked whether the speed of chromaticity change affects discriminability in simulated Mondrian scenes. The results suggest that when the change in surface chromaticities is sufficiently slow, the change is attributed to a global illumination change, even when spatial uniformity is violated.

Complementarily, Nascimento et al. (1996) found that simulated illumination changes over several seconds – linear changes in chromaticity away from D65 in the CIE u'v’ plane - are detectable even when very slow. Although the data are limited and the effects of rate of change are not disentangled from those of magnitude, nonetheless detectability seems to be the same for surface patches in isolation versus surrounded by a Mondrian, and change detection thresholds are lowest along an approximately red-green direction. Both studies also used only a small number of participants (*n* = 2).

Thus, these results suggest that sufficiently slow, continuous changes in surface chromaticity over time may be perceived as illumination changes, regardless of whether these preserve spatial cone-excitation ratios. Yet none of these studies directly assess the speed limits on the detectability of natural changes in illumination over time: how slow are the least detectable changes, and how slow are these relative to natural illumination changes? Further, although these prior studies (Linnell & Foster, 1996; Nascimento et al., 1996) used relatively naturalistic illumination stimuli, deploying gradual chromaticity transitions along the daylight locus with linear temporal profiles akin to natural illumination changes, these were applied only in computer-simulated, small-field images, unlike naturally immersive illumination conditions.

More recently, Kong et al. (2019) directly assessed speed perception of continuous changes in immersive illumination, generated by a five-primary LED lighting system. The individual change stimuli had a periodic temporal profile of semi-linear changes with five full periods per trial. Points of subjective equality (PSE) were determined through comparison of modulations in illumination hue or chroma at different suprathreshold rates in CIELAB space, in a two-interval forced-choice (2IFC) task. Temporal rate PSEs vary with the direction of modulation and the base light chromaticity. For four of five base lights (yellow, purple, blue, and green chromaticities) modulation in hue appears faster than modulation in chroma. Yet because in these periodic stimuli, the temporal profile includes two opposite directions of color change, it is not possible to assess variations in speed perception between the two directions. In addition, modulation rates are calculated assuming the same reference light (daylight metamer D40) as the adaptation white point for all base lights. Yet when experiencing a natural illumination change it is likely that people are already fully adapted to the light at the start of the transition.

Our aim in this study is to examine perceptual sensitivity to natural illumination changes. Therefore, to approximate natural conditions, we use the starting light as the adaptation condition and as the colorimetric white point, and tested individual opposing directions radiating from different base lights along the daylight locus.

Perceptual sensitivity to smooth changes in global illumination over time will depend on the temporal response properties of underlying neuronal mechanisms. Chromatic adaptation, a major contributory factor to color constancy, for example, will set limits on the perceptibility of illumination changes. If adaptation were instantaneous and perfect, even fast changes in illumination may remain below threshold perceptibility. Although there are instantaneous mechanisms that contribute to color constancy (Barbur, 2004), it is generally accepted that chromatic adaptation is a multiphase process, taking place on multiple time scales at multiple levels in the human visual system (Rinner & Gegenfurtner, 2000; Werner, 2014). The time course of chromatic adaptation is also typically measured as the time for color appearance to stabilize after an abrupt change, rather than the phase lag in following smooth changes. A recent study (Spieringhs, Murdoch, & Vogels, 2019) directly compared adaptation progression following abrupt versus smooth changes, and found distinct time constants for the two. After abrupt changes, although most compensation occurs within the first minute of illumination swap (Fairchild & Lennie, 1992; Fairchild & Reniff, 1995) it might take up to 5 minutes to reach full stabilization of color appearance (Gupta et al., 2020; Hunt, 1950; Jameson, Hurvich, & Varner, 1979). More generally, the temporal response properties of chromatic and luminance neuronal mechanisms are characterized by temporal contrast sensitivity functions, with a particular focus on the critical fusion frequency (CFF), or the upper limit at which periodic temporal changes may be followed. In general, CFFs are higher for luminance versus chromatic modulations (Swanson et al., 1987), and vary across individual cone mechanisms as well as higher-order neuronal mechanisms (parvocellular versus magnocellular; Huchzermeyer & Kremers, 2016; Huchzermeyer & Kremers, 2017; Huchzermeyer et al., 2018); CFFs also vary with mean retinal illuminance and stimulus size. It is important to note nonvisual neuronal mechanisms might also influence behavioral responses to temporal changes in illumination. For example, neurons in the suprachiasmatic nucleus of mice are shown to respond to temporal changes in the chromaticity of daylight (Walmsley et al., 2015). Whether such responses exist in the human nonvisual neural pathways and interact with the visual responses is an open question. Here, we focus on the lower limit of perceptibility of aperiodic temporal changes in chromaticity, for large-field, constant luminance stimuli.

In summary, relatively little is known about human visual sensitivity to changes in illumination spectra over time. Illumination changes are usually represented as simplified stimuli with non-natural abrupt changes. The few studies that use sophisticated stimuli with naturalistic changes use only suprathreshold velocities and are not specifically concerned with measuring speed limits of its perception. Yet, a better understanding of how we perceive nonabrupt changes is relevant because smooth transitions in illumination are ubiquitous; natural light, in particular, changes its color temperature continuously throughout the day. People are generally aware of such changes, yet do not seem to perceive such changes as directly as when an unexpected and rapid change in the ambient light condition occurs (e.g. a dark cloud suddenly obstructs the sun). The question we address here is, why do people not perceive the large color temperature changes known to be present in natural daylight? We propose the hypothesis that those changes occur too slowly to be detected.

We, therefore, assess detection thresholds for ecologically relevant temporal changes in illumination chromaticity. Specifically, we determine the minimum detectable chromaticity change of daylight metamers in a fixed time interval, via psychophysical testing, in a naturalistic immersive environment lit with a spectrally tunable illumination system.

## Methods

### The set-up

**Figure 1.**
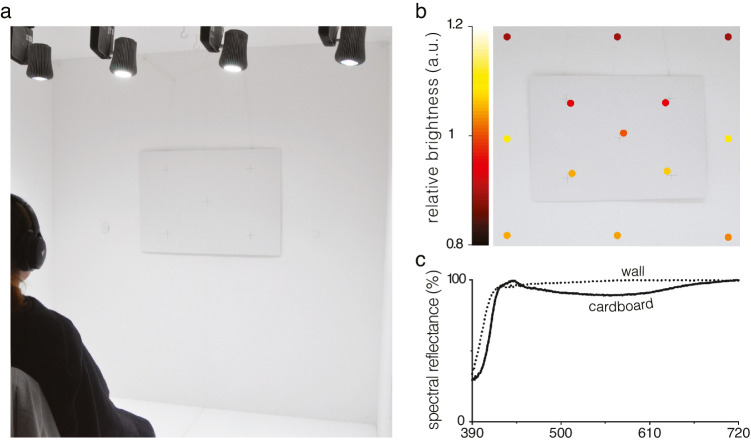
The experimental set-up. (**a**) The participant sat in a lightroom wearing headphones and a black cloth fixating the viewing scene from a distance of about 1.70 m. The enclosure of matte white walls formed an immersive environment. The interior illumination was generated by four spectrally tunable overhead lamps, which produced the smoothest-possible metamer of each desired chromaticity. (**b**) Closeup picture of the viewing scene, constituted by a white matte cardboard (visual angle ≈ 20 degrees × 28 degrees) with five fixation crosses. The overlay of colored dots pseudo-color-codes the relative local luminance with respect to the central measuring point, for 13 positions across the viewing scene and back wall. (**c**) The wall and the cardboard had approximately flat reflectance spectra across the visible spectrum.

Figure 1a shows a participant sitting inside the lightroom at a viewing distance of about 1.70 m. This lightroom consists of a 2 m × 2 m × 2 m enclosure with matte white walls, illuminated by 4 spectrally tunable LED lamps (Ledmotive; www.hi-led.eu) evenly spaced on the ceiling. The spectral power distribution of their outputs is controlled in real-time via computer by setting the weights of the 10 different LED channels in each lamp. In this study, for each desired illumination chromaticity, the weights were optimized using in-house spectral fitting software to produce the smoothest possible, and hence most naturalistic, spectral power distribution. Full details available in previous publications (Finlayson et al., 2014; Pearce et al., 2014).

To ensure the validity of conclusions for global illumination and general applications, both the scene - white matte cardboard subtending 28 degrees × 20 degrees of visual angle, placed on the back wall - and the participants were immersed in the illumination (the only source of light in the room). The inner walls and the cardboard had approximately flat surface spectral reflectance functions in the visible spectrum, with an average reflectance of 96% and 89%, respectively (Figure 1c). Their nonselective spectral reflectances provided a globally uniform illumination chromaticity. Luminance varied smoothly over the walls, but the viewing scene was purposely confined to the most uniform region. Participants were instructed to look at the white cardboard fixating freely between its five evenly spaced fixation crosses. This semi-free movement of gaze reduced visual discomfort and mitigated potential after-image effects. The luminance at the central fixation cross was approximately 95 cd/m^2^, and was kept constant throughout the experiment. The luminance difference between adjacent fixation crosses was on average 6% (and no more than 13%) of the average cardboard luminance (further details in section B. Calibration).

The participant held a black gamepad to enter responses and a black cloth covered his/her clothes. This black cloth protected against potential mutual reflections between the participant's clothes and the white walls and, therefore, helped to maintain congruence with the calibration condition. Some participants wore noise-canceling headphones (TT-BH22UK, Taotronics) as an extra measure against unwanted auditory cues potentially generated by the normal working of the lamps. The lamps were known to produce a slight hissing sound but in a previous study, the hypothesis that the sound could be used as a clue to the specific output illumination was tested and found to be false. In this study, we encountered for the first time a participant who appeared able to capture and interpret very subtle sounds that helped identify changes in the spectral output of the lamps. That particular participant was excluded from the study, and noise-canceling headphones were implemented in all further experimental sessions. In total, headphones were used in 75% of the sessions.

### Calibration

The combined outputs of the lamps were calibrated specifically for this experimental setup, by measuring the spectral basis functions of each LED channel in 50 steps of power level between the minimum and maximum power. The irradiance and illuminance measurements were done at the participant's eye level using an illuminance spectrophotometer (CL-500A Illuminance Spectrophotometer, Konica Minolta), with the room set up the same as in the experiment conditions, including having the black cloth worn by the participants placed in an analogous position. Hence, the effects of mutual illumination between the main surfaces inside the lightroom (white walls, viewing scene, and black cloth) were taken into account in characterizing the light field, so that the spectral power distributions of the generated illuminations were therefore accurately specified at the viewer's eye. The LED channel basis functions obtained in this way served as input to the spectral fitting software used to specify the desired illumination.

Spectral radiance measures were obtained using a CS-2000 Spectroradiometer (Konica Minolta, Nieuwegein, The Netherlands) at 5 locations adjacent to the fixation crosses in the viewing scene and 8 locations of the surrounding back wall. Local luminance measures are presented relative to the central point of the viewing scene (relative luminance of 1 corresponding to 95 cd/m^2^, with the illuminance of 340 lux at the eye), and ranged from approximately 0.9 to 1.1. The lowest levels are found in the upper region and gradually increase to the inferior and side regions in a symmetric fashion. The luminance measures across the viewing scene and back wall are shown in Figure 1b relative to the central luminance.

### Test conditions

**Figure 2.**
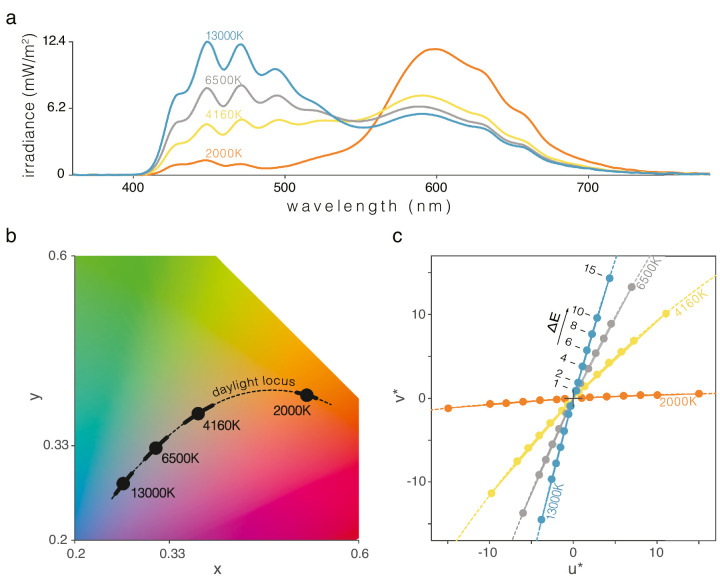
The four base lights tested. (**a**) Irradiance spectra of the 4 base lights, identified by their CCT values: 13,000 K, 6500 K, 4160 K, and 2000 K. (**b**) CIE (x, y) locations of the base lights (black dots). The dashed line represents the daylight locus and the thick black lines emerging from the base points represent the full range of daylight change tested for each base light. The colored background is used for illustration purposes only. These data are available in a more perceptually uniform color space (CIE UCS diagram) in Figure A1, Appendix A. (**c**) Changes were generated with constant luminance in CIELUV uniform color space and assuming the respective base chromaticity as the white point of the color space. The four sets of change vectors (solid lines) and daylight locus (dashed lines) are plotted as different color sets of lines for each white point. Discrepancies between the change vectors and the daylight locus estimates are well below the chromatic discriminability threshold (< 1 ∆E_u*v*_). The colored dots mark the set of end colors for each level of change ∆E: (1, 2, 4, 6, 8, 10, and 15) for both cool- and warm-changes (4 participants in pilot trials used [1, 2, 3, 4, 5, 8, and 15] instead).

Test conditions were smooth, equiluminant changes in global illumination chromaticity over time along the daylight locus. The changes emanated from four starting chromaticities (“base lights”) on the daylight locus: two at the extremes of the locus (with CCTs of 13,000 K and 2000 K) and two near the neutral chromaticities (6500 K and 4160 K; for CIE chromaticity coordinates, see Table 1). For each base light, two directions of change were tested, corresponding to increasing and decreasing CCTs, which we denote here as “cool-changes” and “warm-changes,” respectively. The base light corresponds to both the transition starting point and the adaptation condition, which approximates natural conditions. Their irradiance spectra and location on the daylight locus are illustrated in Figure 2. Base light illuminances were kept constant at a photopic level (approximately 340 lux at eye level) for all stimuli.

**Table 1.** Mean illumination change thresholds and corresponding standard errors for the different base light conditions.

Base lights		Thresholds (∆E CIELUV)			
CCT	CIE (x, y)	Cooler changes	Warmer changes	Mean	Significance level for direction (simple main effects)
13,000 K	0.27, 0.28	4.36 ± 0.37	8.33 ± 0.62	6.35 ± 0.40	**F(1,21) = 38.001, *P* < 0.002
6500 K	0.31, 0.33	4.24 ± 0.36	5.30 ± 0.35	4.77 ± 0.22	NS F(1,21) = 3.672, *P* = 0.276
4160 K	0.37, 0.38	3.94 ± 0.29	2.88 ± 0.20	3.41 ± 0.18	*F(1,21) = 9.596, *P* < 0.02
2000 K	0.53, 0.40	5.91 ± 0.61	3.64 ± 0.34	4.78 ± 0.42	**F(1,21) = 19.665, *P* < 0.002

A total of eight types of illumination transition (4 base lights × 2 directions) were tested, each at seven different velocity levels. These are expressed as the total chromaticity change over the duration of the stimulus, in CIELUV ∆E_u*v*_, as illustrated in Figure 2c.

**Figure A2.**
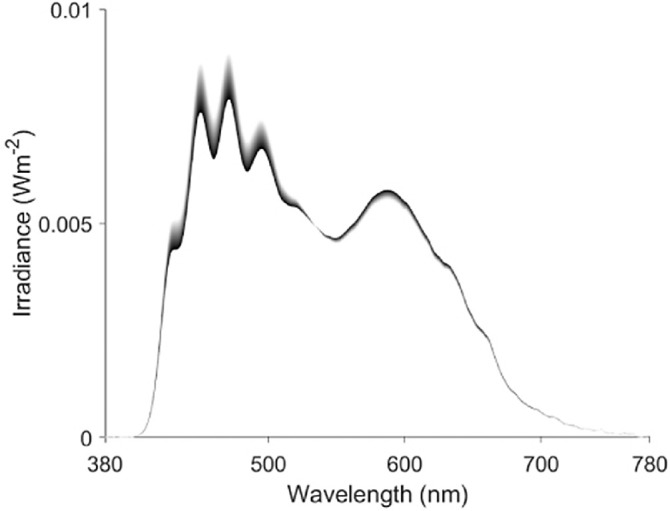
Demonstration of the smooth spectral change corresponding to an example illumination transition (cool-change of the 65,000 K base light corresponding to a color difference of 15 CIELUV units). The spectral basis functions of the constituent steps of change are color-coded from dark to light between the initial spectrum (base light) and the end spectrum.

The duration of each illumination transition stimulus was held fixed at 10 seconds. For each stimulus, the 10 second transition was assembled from 1001 spectra, which were presented in succession to produce a perceptually uninterrupted progression between the start and end chromaticities. Each spectrum in the transition was generated as the smoothest metamer possible for that chromaticity (see Figure A2, Appendix A, for an example transition). The corresponding frequency of change between illuminations within the transition (100.1 Hz) was well above reported frequency thresholds for smoothness perception of linear temporal transitions (Sekulovski et al., 2007). That is, the temporal step size was small enough to make the change appear smooth and without any discernible discontinuities. The small chromatic steps were evenly distributed in a linear interpolation between the start and end chromaticities expressed in CIE (x, y) chromaticity coordinates.

### Design

To measure detection thresholds of illumination changes, the method of constants (MOCs) was implemented with a yes-no task in a one-interval forced-choice (1IFC) trial design (Kingdom & Prins, 2010). The 1IFC yes-no tasks are considered more prone to bias than other task designs with more intervals (Stanislaw & Todorov, 1999). Nonetheless, this is the optimal experimental design given the following considerations. Unlike monitor displays, the global lightroom setting allows only one stimulus to be presented at a time. Where each stimulus must be shown for a long period it becomes impractical to present two or more stimuli in succession and expect the participant to choose between them accurately without any higher-level distorting effects depending on memory or attention. Because here we proposed to focus on slow transitions and therefore needed to use longer durations of stimulus (10 seconds), we opted to use the yes-no 1IFC task instead of a 2IFC.

A series of pilot experiments established the initial set of MOC levels for the first four participants: (1, 2, 3, 4, 5, 8, and 15) ∆E_u*v*_. These levels were slightly adjusted to minimize gaps between levels. The MOC levels used for the remaining participants was (1, 2, 4, 6, 8, 10, and 15) ∆E_u*v*_.

### Procedure

The task required discrimination of smooth temporal changes in illumination without perceived discontinuities, and thus may be considered a modified version of the illumination discrimination task (IDT; Aston et al., 2019; Pearce et al., 2014) in which abrupt transitions in illumination are used. We, therefore, label this task the temporal-IDT (or TIDT).

We opted for monotonic transitions of linear temporal profile starting at the adaptation chromaticity because it better resembles transitions of natural outdoor illumination and allowed us to test opposite chromatic directions independently. In pilot experiments, we tested periodic stimuli with sinusoidal temporal profiles as well, but this type of stimulus proved inadequate for long transitions due to adaptation effects induced across the temporal profile that unreliably altered the perceived colors out of phase with the sinusoidal stimulus, consistent with previous adaptation studies (Bachy & Zaidi, 2014; Zaidi et al., 2012).

At the start of the first session, each participant completed a tutorial program with example trials and standardized verbal instructions via computer-synthesized speech to ensure identical delivery of instructions across participants (list of instructions available in Table A1 at Appendix B). At the end of the tutorial, participants were free to ask questions or repeat the tutorial.

Each participant completed three blocks in one experimental session, with optional breaks of unconstrained length in between. Each block of trials lasted 30 to 40 minutes depending on response times so that each laboratory visit lasted at most 2 hours. One block consists of 84 trials. Each observer completed three blocks per base light, randomly interleaved within experimental sessions, and completed four experimental sessions in total.

**Figure 3.**
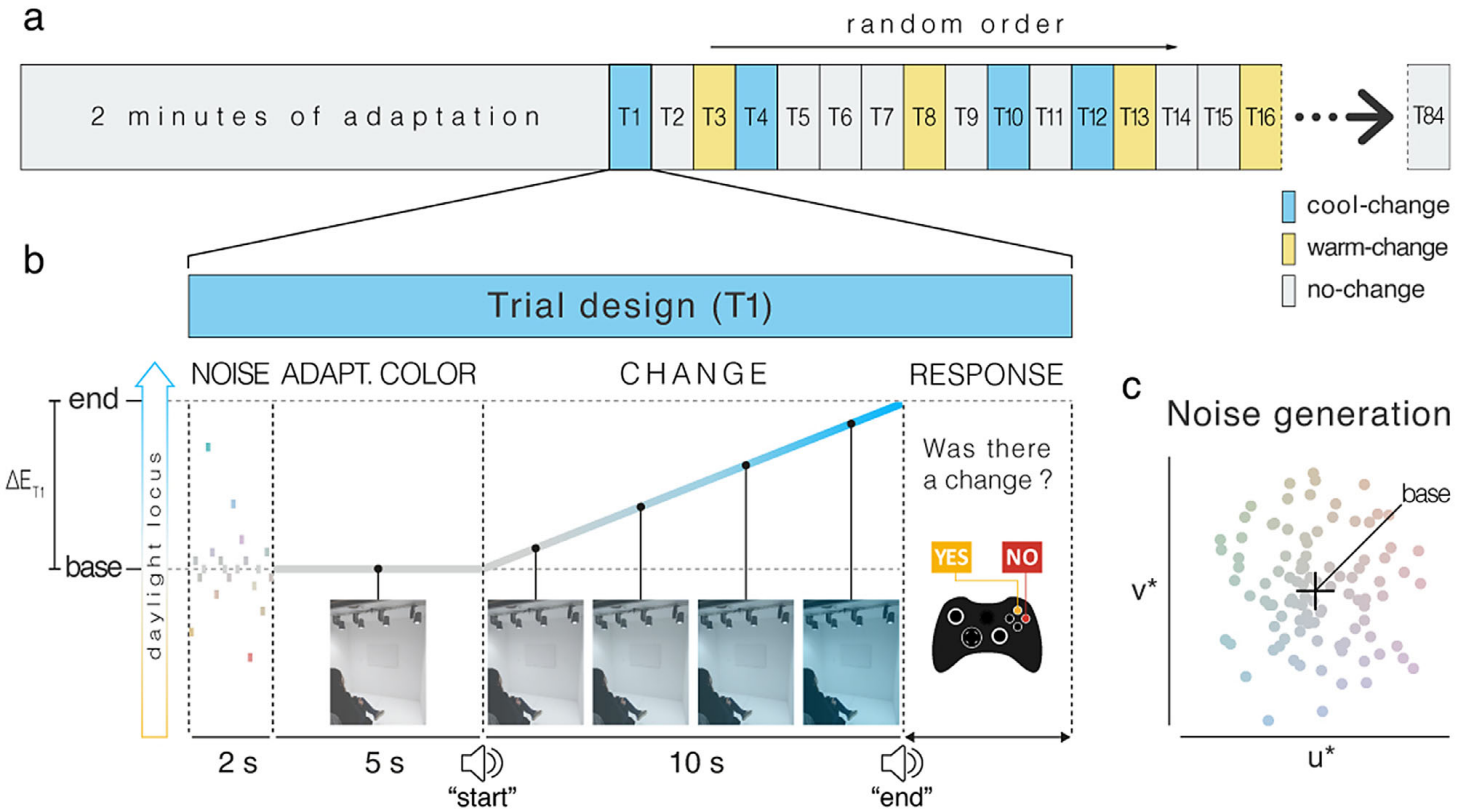
Experimental procedure overview. (**a**) Each block begins with 2 minutes of adaptation to the base color, followed by a random sequence of 84 1IFC trials halved between target-absent trials (no-change) and target-present trials (cool-change and warm-change). (**b**) Trial design of T1 as an example. The structure of the individual trial is composed of 4 parts: NOISE – to mask the chromatic state between trials, each trial began with 2 seconds of chromatic noise stimulus corresponding to a sequence of 20 randomly generated isoluminant lights whose mean chromaticity matched the base chromaticity; ADAPTATION COLOR – 5 seconds of top-up adaptation to the base light; CHANGE – perceptually smooth isoluminant transition between the base and end illumination chromaticity expressed in CIELUV assuming the base color as the white point. The amount of change (∆E) is varied across trials while the duration was held fixed at 10 seconds, and was marked by the sound “start” and “end.” For example, trial T1 contains a change of a certain amplitude ∆E_T1_ in the cool direction, but T3 has a warm-change and T2 has no change at all (∆E = 0); RESPONSE – the last portion of the trial is the response period when the participant is forced to choose between one of two choices “yes, I saw a change” or “no, I didn't see a change.” If the response input is not immediately provided at the start of this period, the noise stimulus is continuously generated until it does. Once input is received the current trial ends and the next trial is automatically initiated. (**c**) Example distribution of the random noise stimuli expressed in CIELUV color space. The noise is randomly generated from normal distribution with a mean value set at the base chromaticity and a standard deviation of three CIELUV units.

Figure 3a illustrates the structure of an individual block of trials. Each block of trials was preceded by 2 minutes of adaptation to the base light under test. Each block of trials was made of a randomly generated sequence of 84 1IFC trials divided equally between target-absent trials and target-present trials, of which 21 were warm-change and 21 cool-change, with 3 trials for each of the 7 MOC levels.

The trial design is exemplified in Figure 3b for a cool-change trial. In each trial of the experiment, the observer was first exposed to 2 seconds of chromatic noise (rapid, random, small changes in global illumination chromaticity), followed by 5 seconds of adaptation to the base light. Then, within a fixed period of 10 seconds marked by computer-generated auditory cues (“start” and “end”), a smooth chromatic transition away from the adaptation chromaticity occurred in the ambient illumination. Thus, the duration of the transition was fixed while the amount of change, expressed in CIELUV as ∆E_u*v*_, varied across trials for threshold estimation. The participants were instructed to pay close attention during each trial only to changes that occurred between the sounds “start” and “end.”

The 2-second chromatic noise stimulus served the purpose of masking the chromatic illumination state between trials (see Figure 3c). It was an isoluminant sequence of 20 randomly generated spectra whose mean chromaticity matched the base chromaticity, with SD of 3 CIELUV units. Pilot testing showed this value was enough to mask the chromatic stimuli between trials without causing afterimages, which might influence the main stimulus (the smooth chromatic change). The 5 seconds of top-up adaptation in each trial was used to help ensure mean adaptation to the base light chromaticity through the full extent of the block. Because the illumination transitions were so long (10 seconds), the duration of the top-up adaptation periods was set to the minimum possible that prevented disruption of the mean adaptation level by the larger-excursion trials, yet also allowed completion of the block without inducing fatigue. Presenting cool- and warm-change trials in the same block and testing each base light with 3 separate blocks of trials, each with its own 2-minute adaptation period, mitigated against this potential effect as well.

Participants were also allowed to pause the experiment at the end of any trial to rest their eyes for as long as they needed to outwait any visual discomfort or after-images, which were infrequent but possible depending on the participant's susceptibility to these phenomena. During the pause mode, the participants were exposed to the adaptation light.

The response period begins after the “end” sound. During this unconstrained period, the noise stimulus was produced until the participant's response triggers the start of the next trial. Participants were usually fairly quick to respond, and the response time was often zero as most participants would determine their responses shortly before the response period started. Figure 3d schematizes the question-response procedure of the task. The participants were trained during the tutorial to indicate whether they saw a change in illumination by pressing “yes” or “no” on the gamepad.

### Data analysis

The analysis procedure was applied to the combined data from the 3 blocks of trials for each base light, for a total of 252 trials for each base light (63 target-present trials for each direction, and 126 target-absent trials).

The thresholds for the two illumination-change directions were computed separately from yes-no data via the Palamedes toolbox (Prins & Kingdom, 2018) in MATLAB (MathWorks, Inc., Natick, MA, USA) by implementing the bias correction method recommended for yes-no tasks (Kingdom & Prins, 2010). From the proportions of false-alarms and hits the appropriate Palamedes routines were used to compute the discrimination index d' (Swets, Tanner, & Birdsall, 1961) for each stimulus level, which was converted to proportion correct Pc assuming an ideal value of zero (no response bias) for the participant's criterion C. This provides a bias-corrected Pc estimate, termed Pc_max_, to which the Quick function (Quick, 1974) was fitted via maximum likelihood estimation. The threshold derived from the Pc_max_ psychometric function was the parameter of choice for the main unbiased measure of psychophysical performance.

To test slow changes relatively long trials were required, which in turn imposed limitations on the number of trials that allow keeping task demands within reasonable bounds for attention. These restrictions resulted in cases of negative d’ (13% of the data points). These correspond to MOC levels where the participant pressed “yes, I see” proportionally less often on target-present trials than on target-absent trials. Such responses may occur through sampling error or response confusion (Stanislaw & Todorov, 1999). The latter is unlikely, as all participants underwent mandatory practice trials to make sure they understood the controls before the start of the experiment. Sampling error seems the more likely cause as it increases inversely with the number of trials for each MOC level but is also influenced by the difficulty each participant experiences in performing the task. The relatively small number of trials per MOC level combined with the task demands on attention was likely to increase sampling error. Because these cases tended to occur below threshold levels, the corresponding points were adjusted by clipping pF to the value of pH, as this approach assumes that the real performance was near chance level.

### Participants

The experiment was carried out by 22 healthy participants (5 men and 17 women, mean age of 24 ± 5 years). The participants were drawn from local and international populations (10 nationalities) of Newcastle upon Tyne. An exclusion criterion for ages above 40 years was set, to minimize the influence of age-related variations in attention for this relatively demanding task.

Demographic data, including age, sex, race, and nationality were recorded together with self-reported information about general and visual health. Participants who were prone to epileptic seizures were excluded. All participants had normal or corrected-to-normal visual acuity (self-report) and normal trichromatic color vision, confirmed by in-laboratory testing with the Ishihara Color Test 38 plates edition (Ishihara, 1977) and the Farnsworth-Munsell 100-Hue test (Farnsworth, 1957). In addition, each participant completed the Morning-Evening Questionnaire Self-Assessment version (MEQ-SA; Terman & Terman, 2005), made available by the Center for Environmental Therapeutics (n.d.), to determine whether their daylight routine was skewed toward morning or evening and estimate the time of day each participant is usually exposed to daylight.

Participants received cash compensation for their time.

### Ethics

The study was reviewed and approved by the Faculty of Medical Sciences Research Ethics Committee, part of Newcastle University's Research Ethics Committee (approval 7089/2018), which includes members internal to the faculty, as well as one external member. Committee members must provide impartial advice and avoid significant conflicts of interest. The experiments were performed in accordance with the tenets of the Declaration of Helsinki. A detailed information sheet was provided, and written consent was received from all participants prior to participation in the study.

## Results

**Figure 4.**
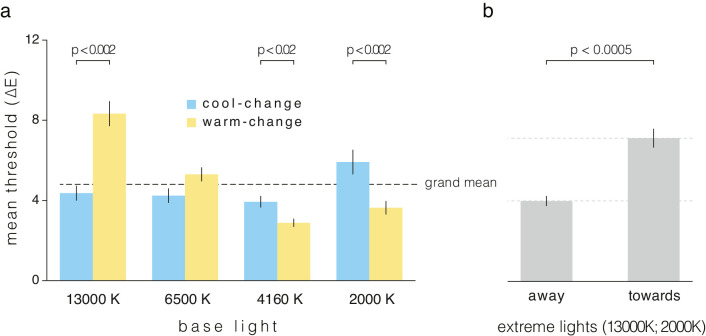
Detection thresholds for chromatic illumination changes. (**a**) Threshold values in ∆E CIELUV (for the fixed period of 10 seconds) averaged across participants (*N* = 22) for each test condition (cool- and warm-change) for each of the base lights. The grand mean estimated across all conditions is shown by the horizontal dashed line. Statistically significant differences between chromatic directions of change are indicated with the respective *P* values (see Table 1). (**b**) Comparison between mean thresholds of changes away and toward neutral chromaticities, estimated from the two most extreme lights 13,000 K and 2000 K. Error bars denote the corresponding standard error.

Figure 4a presents the average thresholds estimated across the 22 participants for each test condition (2 illumination-change directions per base light). The results ranged from 14.7 to 1.6 ∆E CIELUV units. These maximum and minimum values correspond, respectively, to the 13,000 K warm-change and 4160 K warm-change conditions. These color difference values, occurring for the fixed period of 10 seconds correspond to velocity thresholds of 1.47 ∆E/s and 0.16 ∆E/s, respectively.

A 4 × 2 repeated measures ANOVA revealed that there is a significant interaction between base light and chromatic direction of illumination change, F(3, 63) = 40.458, *P* < 0.001. Furthermore, there is also a significant main effect of base light, independent of the change direction, F(3, 63) = 24.341, *P* < 0.001. The main effect of illumination-change direction is not significant, F(1, 21) = 1.315, *P* = 0.264. (In all cases, sphericity was violated with ε < 0.75, so Greenhouse-Geisser corrections were performed.)

Further examination of the main effect of base light shows that all 6 pairs of mean thresholds are significantly different, except for 6500 K vs. 2000 K. In particular, the mean threshold for 13,000 K is significantly higher than for all other lights (*P* < 0.005); for 6500 K, significantly lower than for 13,000 K and higher than for 4160 K (*P* < 0.005); and for 4160 K, significantly lower than all other lights (*P* < 0.05 for 2000 K; see Table 1 for mean thresholds). All significance tests are Bonferroni corrected for multiple pairwise comparisons. To assess the influence of the base light on the effect of illumination-change direction, we performed post hoc analyses on thresholds for each base light (simple main effects analysis of illumination-change direction; see Table 1: across-column comparison within each row). Illumination-change direction has a significant effect on thresholds for all base lights except for 6500 K (for significance levels see Table 1). In each case, the post hoc tests are Bonferroni-corrected for multiple comparisons (i.e. *P* values for significance are multiplied by 4 for the number of light conditions within which the illumination-change directions are being compared).

The far-right column shows Bonferroni-corrected significance levels of the threshold difference between cool- and warm-changes per base light.

For all three base lights where the difference between illumination-change directions is significant, the threshold for the direction toward 6500 K (the nominal neutral chromaticity; i.e. the chromaticity closest to neutral of our set of chromaticities) is higher than in the opposite direction. That is, for cooler lights, the thresholds are lowest for cooler changes, whereas for warmer lights, thresholds are lowest for warmer changes. These opposing differences in thresholds for base lights on opposite sides of neutral explain the lack of a main effect of illumination-change direction expressed as cooler or warmer. Yet, if we instead express the illumination change as toward or away from neutral, the overall effect of direction is clear. The largest threshold difference between directions occurs for the most extreme lights, 13,000 K and 2000 K. Combining the toward- and away-from neutral data for these two lights (i.e. 13,000 K-warmer and 2000 K-cooler; 13,000 K-cooler and 2000 K-warmer), we find a significant effect of illumination-change direction, F(1,43) = 53.537, *P* < 0.0005 (see Figure 4b). Thus, changes in illumination chromaticity toward neutral are hardest to detect, for non-neutral reference lights. The corresponding values are 4.00 (± 0.25) and 7.12 (± 0.47) in ∆E CIELUV units, for away and toward, respectively.

Between participants, MEQ scores correlated significantly with age (rho = −0.435, *P* < 0.05), yet neither MEQ score nor age predicted thresholds within or across base light conditions.

## Discussion and conclusions

We measured the visual discriminability of smooth changes over time in global illumination that mimicked typical changes in natural daylight chromaticity. For four distinct starting illumination CCTs on the daylight locus, we measured detection thresholds for linear rates of change in chromaticity only, in two directions each, toward cooler or warmer CCTs. In an immersive setting with no explicit objects or spatial features, participants were able to reliably perceive these temporal changes in global illumination. Sensitivity to the rate of change varied significantly with both the starting chromaticity and the chromatic direction of change, with the highest sensitivity occurring around neutral starting chromaticities, where rates as low as 0.16 ∆E/s were detectable.

Thresholds were largest for the most extreme chromaticities, with mean thresholds over both directions largest for the coolest illumination CCT tested (13,000 K), followed by the 2000 K illumination, and the two near-neutral lights, 6500 K and 4160 K. The difference between warm- and cool-changes was significant for all 4 base lights, except 6500 K. In addition, direction had stronger effects for the two most extreme CCT levels used (13,000 K and 2000 K) and the effects for those lights were opposite.

For the base lights where the difference between directions is significant, the threshold for the direction toward 6500 K (the nominal neutral chromaticity) is higher than in the opposite direction. These opposing differences in threshold for base lights on opposite sides of neutral result in no overall main effect of illumination-change direction. Thus, sensitivity depends on base light CCT and chromatic direction in a cross-over interaction: cool-changes become less noticeable for progressively warmer base lights and vice-versa. In Figure 4a, changes toward nominal neutral chromaticities seem to be the main driver for the direction related differences. This observation is further supported by the analysis in Figure 4b, which shows that the difference between the grand mean (4.82 ∆E) and “toward” thresholds (7.12 ∆E) is about three times larger than that for “away” thresholds (4.00 ∆E). These considerations indicate that the point of peak sensitivity occurs near the achromatic region of the daylight locus.

Although the direction of change has a significant effect at local levels (for base lights 13,000 K, 4160 K, and 2000 K), it does not overall. The marginal means of warm- and cool-change thresholds over all base lights (5.04 ∆E and 4.61 ∆E, respectively) did not differ significantly, because results of opposite directions at opposite ends of the CCT continuum counteract each other, leading to the crossover interaction referred to above. This lack of overall significant effect highlights the importance of testing continuous changes in a linear temporal profile instead of a periodic one. For example, the base light conditions with CCTs 6500 K and 2000 K have the same marginal mean thresholds (4.8 ∆E). If a sinusoidal stimulus had been used instead, illumination discrimination sensitivity would have been deemed the same at these two base lights. Thus, previous data on the perception of smooth illumination changes collected with periodic stimuli (Kong et al., 2019) might have overlooked relevant effects of chromatic direction.

Previous studies of illumination discrimination find that abrupt discontinuous changes in illumination chromaticity toward “blue” are less easily discriminated than changes in other chromatic directions (Pearce et al., 2014), whether or not spatial relationships between surface reflectances remain stable (Radonjić et al., 2018), and, on average, across multiple starting (reference) illumination chromaticities (Aston et al., 2019). Here, we find that this “blue bias” depends definitively on the starting illumination chromaticity: only for the two base lights warmer than D65 (6500 K) are changes toward cooler daylight temperatures less easily discriminated than changes in the opposite direction. For the coolest base light, CCT 13,000 K, changes toward warmer daylight temperatures have higher discrimination thresholds than in the opposite direction. This finding is consistent with the pattern of thresholds reported previously for discontinuous illumination changes (Aston et al., 2019). Although, on average, thresholds are higher for “bluer” directions, the opposite is true for the “blue” reference light, for which thresholds are highest in the “yellower” direction (Aston et al., 2019). For red and green reference lights, Aston et al. (2019) also found that changes away from the reference chromaticity toward neutral are less easily discriminated than changes in the opposite direction, away from neutral. Thus, the overall pattern of thresholds in Aston et al. (2019) suggests a more general neutral bias in illumination discrimination, not exclusive to the daylight locus.

The neutral bias reveals itself in our results with the reversal of threshold differences on either side of the neutral chromaticity region, where the effect of direction is not significant and the largest sensitivity to illumination change is found. One interpretation of the neutral bias is that the human visual system encodes a neutral daylight illumination prior (i.e. that it stores a representation of natural illumination distributions in which neutral illuminations are more likely). Although this explanation is plausible, it remains to be tested systematically. Another interpretation is that the human visual system has evolved to become less sensitive to illumination changes that are more frequently encountered in the natural environment. If so, we may ask whether smooth temporal changes in daylight are more common toward rather than away from neutral chromaticity. This question remains open and is subject to further analysis. The question of whether the human visual system is optimized to follow or discount illumination changes along the daylight locus (“blue-yellow”) versus orthogonal directions (“red-green”) is also not specifically addressed by this experiment but may readily be addressed in further experiments with this technique.

**Figure A3.**
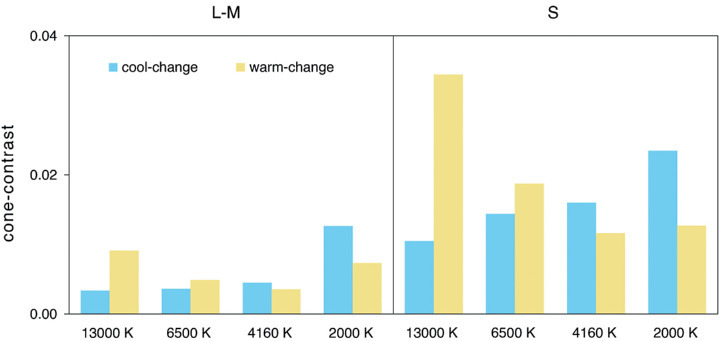
Detection thresholds of Figure 4a re-expressed in cone-contrast units for the L-M and S components of the DKL color space. Threshold values were averaged across participants (*N* = 22) for each test condition (cool- and warm-change) for each of the base lights and were estimated assuming the corresponding base light as the white point.

More fundamentally, the neutral bias here – higher detection thresholds for changes toward neutral – may be explained by properties of low-level chromatic mechanisms. Given the known asymmetry in the neural architecture and physiological properties of the S-cone ON and OFF channels (Chichilnisky & Kalmar, 2002; Shinomori, Spillmann, & Werner, 1999; Zaghloul, Boahen, & Demb, 2003) it is important to ask whether this asymmetry might explain the threshold differences between change-directions for any base light. We, therefore, re-expressed all illumination change thresholds in terms of individual cone-opponent and total cone-contrasts between the base light and transition lights (in DKL cone-contrast space; Derrington, Krauskopf, & Lennie, 1984). The pattern of thresholds remains the same, with higher amounts of both S and L-M cone-contrasts required for detection of warm-changes at the cooler temperatures, and vice-versa (see Figure A3, Appendix A). The neutral bias here is therefore not explained by differential sensitivities to S-cone increments versus decrements.

Other reported asymmetries in chromatic discrimination of static stimuli also do not readily explain the neutral bias seen here. For different levels of background S-cone stimulation, qualitatively corresponding to different global illumination chromaticities, detection thresholds for small targets against large uniform backgrounds change symmetrically in the blue and yellow directions (Krauskopf & Gegenfurtner, 1992). The asymmetry between detection thresholds for hue versus saturation changes in small segmented disks against large uniform backgrounds (Danilova & Mollon, 2016) does not predict the thresholds observed here, because for each base light the warmer versus cooler changes lie along the same line in cone-contrast space.

The mechanisms underlying the observed asymmetry in detection thresholds are therefore as yet unknown. Whether the pattern of thresholds may be entirely explained by differences in temporal response properties of low-level chromatic mechanisms remains an open question. At the very least, the temporal properties of low-level mechanisms of chromatic adaptation must constrain the perceptibility of global changes in illumination chromaticity. Yet the slowest detectable temporal changes in illumination chromaticity that we find here are not only much lower than the rates of illumination change previously simulated (no less than 2 ∆E/s - according to calculations from the available data on the previous temporal illumination studies; Kong et al., 2019; Linnell & Foster, 1996; Nascimento et al., 1996; Walmsley et al., 2015), but also lower than the lower limits of temporal frequency typically explored in fundamental temporal contrast sensitivity measurements (Huchzermeyer et al., 2018). It is therefore difficult to relate the results directly to measurements of low-level chromatic mechanisms. The results suggest, though, that low-level chromatic mechanisms might differ in their adaptation response properties at low temporal rates of change.

Differences in overall adaptation to the base light chromaticity might also contribute to differential sensitivity. These cannot be ruled out, but are unlikely. In a separate study (Gupta et al., 2020), we found that the parameters of the exponential progression of adaptation, as measured by the change in achromatic settings over 5 minutes, did not depend significantly on global illumination chromaticity, even for extreme chromaticities. Given the long exposure durations (30–40 minutes) of each trial block, it is therefore unlikely that differences in completeness of adaptation underlie the significant variation in detection threshold patterns between base lights.

**Figure 5.**
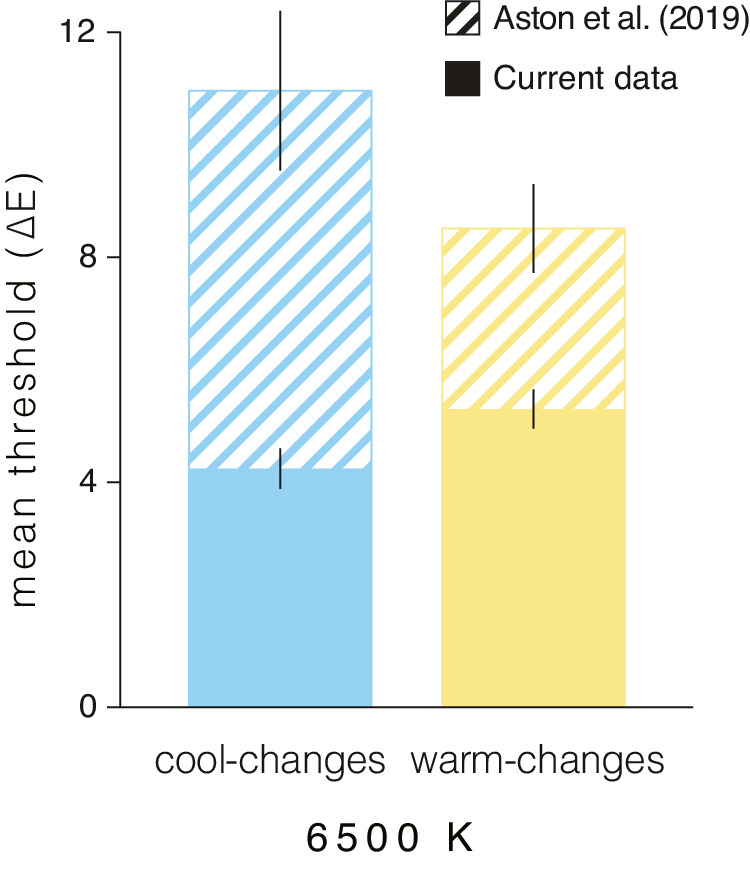
Comparison of illumination discrimination thresholds for cool- and warm-changes between the current data and the previous data collected for Mondrian scenes with abrupt illumination changes (Aston et al., 2019). The comparison is made for the only light in common, a daylight metamer with CCT of 6500 K (commonly known as D65). Error bars denote the corresponding standard error.

This study differs from traditional studies of color constancy in examining sensitivity to changes in illumination spectra rather than constancy of surface color appearance under such changes. Because the scene is effectively unarticulated, consisting of a white surface against a white wall, no information is provided from spatial cone-excitation ratios, and thresholds were expected to be smaller than in articulated scenes in which the stability of spatial cone-excitation ratios masks the illumination change (Foster et al., 1997). In a previous study using the illumination discrimination task, Aston et al. (2019) used highly articulated scenes and obtained larger thresholds than the current experiment for a chromaticity condition common to both (i.e. 0.31, 0.33; CIE (x, y)), corresponding to the 6500 K base light. Figure 5 compares the corresponding sets of results from both studies. For chromatic directions that closely approximate our cool- and warm-changes, Aston et al. (2019) found thresholds of 11.0 ± 1.4 and 8.5 ± 0.8 ∆E CIELUV units, respectively. These values are about twice as large as those found here for smooth cool- and warm-changes axes (5.3 ± 0.35 and 4.2 ± 0.36, respectively). There are additional experimental differences besides scene articulation worthy of note. Adaptation conditions differed: in Aston et al. (2019) the initial adaptation period was to the dark rather than the reference light, but the reference light was presented for 2 seconds on each trial and would have provided top-up adaptation similar to our 5 seconds of in-trial adaptation. In addition, Aston et al. (2019) used a 2IFC trial design requiring memory retention of the reference stimulus across a blank interval. Although there is consensus that memory affects color representation, its specific effects are not clear (Allred & Olkkonen, 2015), but it is likely that memory demands would decrease sensitivity (Aston et al., 2019).

The smallest chromaticity difference discriminated by participants in this experiment was approximately 2 ∆E_u*v*_ units, which is substantially larger than estimates of the largest chromaticity difference (at fixed luminance) that occurs in real daylight, for the same period of 10 seconds. For example, analysis of the dataset of daylight measurements reported by DiCarlo & Wandell (2000) indicates a maximum change of 0.3 ∆E _u*v*_ over 10 seconds, assuming the whole range of daylight chromaticities as possible CIELUV white points. Chromaticity changes of daylight over time, thus, are likely to be under threshold detectability, enabling observers to maintain a stable perception of outdoor illumination, at least where there are no concomitant overall illuminance changes. The perceptual limitations on sensitivity to smooth temporal changes in illumination may, therefore, contribute to color constancy under natural daylight. Further analysis of environmental constraints on sensitivity to illumination change is currently underway.

This type of study is made possible by contemporary technology that enables fine control of global illumination. Yet there are still technical limitations that hinder research efforts. For example, there is no widely used perceptually uniform brightness measure that allows control of perceived brightness across lights of different saturation levels (Koenderink, van Doorn, & Gegenfurtner, 2018). In our experiment, a fixed lux level was used across all stimuli, but some participants reported differences in perceived brightness between base lights, especially for the two most extreme lights (“the warmest condition feels slightly darker than the coolest condition”). Nevertheless, participants did not report this effect for spectra within changes tested for each base light and it did not seem to provide unwanted clues.

Last, a comparison may be made with sensitivity to spatial gradients. The human visual system is designed to discount low-frequency smooth spatial changes in illumination (Land, 1977). The results here demonstrate that the visual system similarly discounts slow, smooth changes in illumination over time. The perceptual mechanisms for analyzing spatial and temporal characteristics of a natural scene may be analogous, or even entangled. According to Linnell & Foster (1996), scene changes that preserve spatial cone ratios are more likely to be perceived as illumination changes but if the change in cone ratio is smooth and slow enough, it might go undetected. We speculate that to be perceived as an illumination change, the scene change should have certain temporal characteristics as well as spatial characteristics. Our results show that those temporal characteristics depend on the chromatic direction of change.

In summary, we found that detection thresholds tend to be higher for illumination changes directed toward neutral chromaticities. This neutral bias is consistent with previous results found for stimuli with discontinuous changes (Aston et al., 2019), suggesting that the bias is not exclusive to the temporal profile of change. Yet differences in the absolute thresholds between these studies indicate that temporal parameters of the change stimuli might influence these effects. In addition, other factors, such as the level of scene articulation, memory demands, and adaptation state, also may affect perception of illumination changes and consequent effects on surface color appearance and constancy.

## Figures and Tables

**Figure A1.**
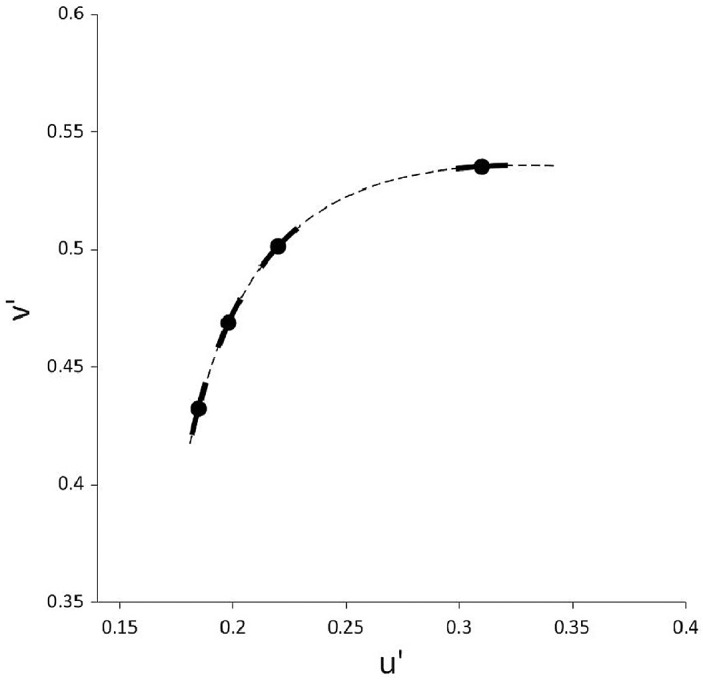
Data of Figure 2b represented in the CIE UCS diagram. The CIE u’ v’ chromaticities of the four base lights correspond to the black dots. The dashed line represents the daylight locus and the thick black lines emerging from the base points represent the full range of daylight change tested for each base light.

## Appendix B

### Tutorial

To ensure identical delivery of instructions across participants during the tutorial, standardized verbal instructions were delivered by computer-synthesized speech. Table A1 lists these instructions in order.

**Table A1.** List of standardized verbal instructions delivered by computer-synthesized speech during the tutorial.

Standardized verbal instructions	
1.	Please keep your gaze on the scene, and never look directly at the lights.
2.	Before the test there will be an adaptation period of about 2 minutes. During this period, you must keep looking at the scene.
3.	After that, the test will start automatically.
4.	During the test the illumination may present some changes.
5.	Your task is to pay close attention to changes that occur in each trial between the word “start” and the word “end.”
6.	Example. *^1^
7.	Did you feel a change in the illumination between the sound “start” and “end”?
8.	If Yes, press Y.
9.	If No, press B.
10.	Practice trials. *^2^
11.	You can also ask for a pause by pressing Start, before pressing Y or B.

*^1^ One trial with an obvious change was shown.

*^2^ Four trials were executed with response feedback: “correct” or “incorrect.”
